# Focal hand lesions: review and radiological approach

**DOI:** 10.1007/s13244-014-0334-4

**Published:** 2014-05-17

**Authors:** Chau Hung Lee, Ankit Tandon

**Affiliations:** Tan Tock Seng Hospital, 11 Jalan Tan Tock Seng, Singapore, 308433 Singapore

**Keywords:** magnetic resonance imaging, hand, wrist, soft tissue neoplasms, bone neoplasms

## Abstract

Focal hand lesions are commonly encountered in clinical practice and are often benign. Magnetic resonance (MR) imaging is the imaging modality of choice in evaluating these lesions as it can accurately determine the nature of the lesion, enhancement pattern and exact location in relation to surrounding tissues. However, while MR features of various soft tissue lesions in the hand have been well described, it is often still difficult to differentiate between benign and malignant lesions. We review the MR imaging features of a variety of focal hand lesions presenting at our institution and propose a classification into “benign”, “intermediate grade” (histologically benign but locally aggressive with potential for recurrence) and frankly “malignant” lesions based on MR findings. This aims to narrow down differential diagnoses and helps in further management of the lesion, preoperative planning and, in cases of primary malignancy, local staging.

*Teaching Points*

• *Hand lesions are often benign and MR is essential as part of the workup.*

• *MR features of various hand lesions are well described but are often non-specific.*

• *Certain MR features may help for the diagnosis but histological examination is usually required.*

• *We aim to classify hand lesions based on MR features such as margin, enhancement and bony involvement.*

• *Classifying these lesions can help narrow down differential diagnoses and aid management.*

## Introduction

Most soft tissue lesions in the hand are benign [1]. Imaging is often required to determine the nature of the lesion. Plain radiography has limited utility but is useful in demonstrating calcification. Ultrasound is a cheap and relatively quick method for determining the cystic or solid nature of the lesion. Magnetic resonance (MR) is the imaging modality of choice as it can accurately determine the nature of the lesion, enhancement pattern and exact location in relation to surrounding tissues given its high contrast and spatial resolution. However, while MR features of various soft tissue lesions in the hand have been well described, preoperative diagnosis of these lesions is often difficult, and even distinguishing benign from malignant lesions remains challenging. We review the MR imaging features of a variety of hand lesions presenting at our institution and propose a classification into “benign”, “intermediate grade” (histologically benign but locally aggressive with potential for recurrence) and frankly “malignant” lesions based on MR findings. This aims not just to narrow down differential diagnoses but also to help in further management of the lesion in terms of preoperative planning, and for cases of primary malignancy, local staging and prognosis.

## Imaging technique

At our institute, MR imaging is performed on a 1.5- or 3-T scanner. Several technical factors need to be considered to get the best images of the hand and wrist. Important technical factors to consider are patient positioning, choice of coil and sequences. Based on the lesion location and extent of coverage required, patients are scanned using an extremity, wrist or best-fit surface coil. Whenever high-resolution imaging using thin slices and a small field of view (FOV) is critical, surface coils are preferred.

Patients are routinely scanned in the supine position with arm by the side. Sometimes patients are scanned in the “superman” position, with the patient lying prone and the arms above their head. Meticulous positioning is important to bring the region of interest to isocenter. When the region of interest is close to the periphery of the coil, auto shimming is used. Skin markers are used to localise small lumps.

The routinely used sequences at our institute include T1-weighted (T1w) sequences in the axial and coronal planes, T2-weighted fat-saturated (T2w-FS) or short tau inversion recovery (STIR) sequences in the axial plane and T2-weighted (T2w) sequences without fat saturation in either the sagittal or coronal plane. A gradient-echo (GRE) sequence is also acquired, which is particularly useful if a vascular lesion or giant-cell tumour of the tendon sheath is suspected. Post-contrast T1w-FS sequences with intravenous gadolinium compounds are acquired in all patients unless contraindicated. Routine FOV of 16–20 cm, slice thickness of 4.0 mm and matrix of at least 512×256 is used, although when higher resolution imaging is critical, a smaller FOV of 8–12 cm and thinner slice thickness of 1.5–3.0 mm are preferred.

## Benign lesions

### Ganglion cyst

**Fig. 1:**
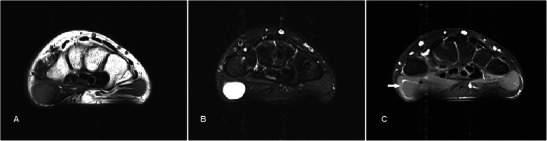
Ganglion cyst in a 24-year-old female who presented with a slowly growing, firm, painless hand lump for about 6 months. (**a**) T1w sequence shows a smooth, well-circumscribed lesion within the thenar musculature of homogeneous low signal. (**b**) The lesion is hyperintense on T2w-FS sequence. No invasion into adjacent structures is seen. (**c**) There is minimal rim enhancement with no significant internal enhancement (arrow)

Ganglion cysts are the most common lumps encountered in the hand and wrist region [2]. They tend to occur in young adults and are three times more frequent in females. They are thought to represent degeneration of connective tissue caused by chronic irritation [3]. The most common location is in the dorsum of the wrist where they usually arise from the scapholunate joint. Less typical sites include the volar aspect of the wrist from the radio-scaphoid or scapho-trapezial joint, at the metacarpophalangeal joint in relation to flexor tendons and distal interphalangeal joints [4]. MR shows a well-circumscribed unilocular or multilocular lesion of fluid signal, although the signal may vary depending on the amount of proteinaceous contents (Fig. 1). Mild rim enhancement of the capsule may be seen, but there is usually no enhancement of internal contents. Differential diagnoses include synovial cysts and other cystic lesions such as epidermal cysts.

### Epidermal cyst

**Fig. 2:**
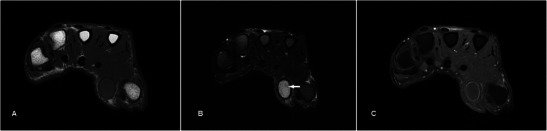
Epidermal cyst in a 42-year-old male presenting with a lump over the palm of about 10-year duration. (**a**) T1w sequence shows a low-signal, smooth, well-circumscribed subcutaneous lesion superficial to the thenar musculature. (**b**) The lesion is hyperintense on T2w-FS sequence with faint low-signal foci within indicated squames (arrow). A thin hypointense capsule is seen. (**c**) Faint rim enhancement is seen with no significant internal enhancement

Epidermal cysts are common hand lesion resulting from proliferation of epidermal cells within a confined space in the dermis. They can be congenital, a result of occlusion of the follicles by adjacent inflammation or tumour, or associated with human papilloma virus infection [5]. True epidermal cysts result from implantation of epithelial squames into the dermis because of trauma. MR shows a well-circumscribed lesion of fluid signal in the dermis or subdermis (Fig. 2). However, signal on the T2w sequence may be variable depending on the amount of internal keratin debris [6]. Rim enhancement can be seen. Lack of central enhancement is key to distinguish epidermal cysts from more sinister lesions such as neurogenic tumours or sarcomas, particularly if heterogeneous signal is present because of internal debris. They may also be mistaken for ganglion cysts if located close to the joint or tendon sheath.

### Fibroma of the tendon sheath

**Fig. 3:**
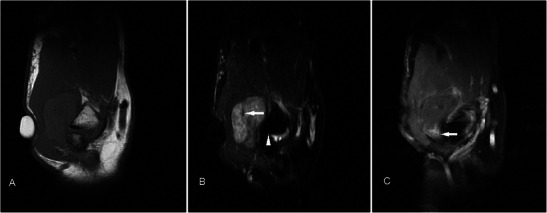
Fibroma of the tendon sheath in a 51-year-old female presenting with a 6-month history of a lump in the left hand over the thenar eminence near the first metacarpal base. (**a**) T1w sequence shows a lobulated mass deep to the thenar musculature. It is slightly hyperintense to muscle, which is unusual. (**b**) The lesion is heterogeneously hyperintense on T2w-FS sequence with low signal areas indicating a fibrous component (arrow). The lesion abuts but does not involve the trapezium (arrowhead). (**c**) A small eccentric focus of enhancement (arrow) is seen with no other areas of significant internal enhancement

Fibromas of the tendon sheath (FTS) are uncommon lesions thought to be a reactive fibrosis. It has a peak incidence at 20–50 years old, is three times more common in males, and 82 % of them occur in the hand and wrist region [7, 8]. The low signal on all MR pulse sequences expected of a fibrous lesion is not always seen. FTS are generally well defined and of low signal on T1w sequence. Signal intensity on T2w sequence and contrast enhancement is more varied and heterogeneous (Fig. 3), likely reflecting varying proportions of fibrous and cellular tissue [9]. Differential diagnoses include more sinister lesions such as soft tissue sarcomas and giant cell tumours of the tendon sheath (GCTTS). FTS and GCTTS are believed to represent two end points of a single clinicopathological entity based on histological studies, the former with a predominance of myofibrolastic markers while the latter with a predominance of macrophage-related components [10]. They can be differentiated based on certain imaging features. On GRE sequence, GCTTS usually demonstrates susceptibility artefacts, which are absent in FTS. Bony scalloping is also often seen in GCTTS but rare in FTS, and while both are most commonly located in the hand, GCTTS is more likely to be found in the feet compared to FTS [9].

### Focal nodular synovitis

**Fig. 4:**
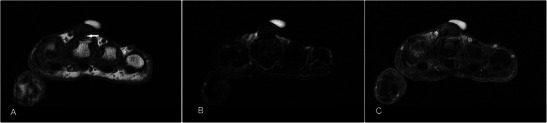
Focal nodular synovitis in a 62-year-old male presenting with a painless lump over the dorsal aspect of the right middle finger for a few months. (**a**) T1w sequence shows a lobulated, homogeneously low-signal subcutaneous nodule over the dorsal aspect of the third metacarpophalangeal joint abutting the extensor tendon (arrow). (**b**) The lesion is also of low signal on T2w-FS sequences. No bony destruction is seen. (**c**) There is no significant contrast enhancement

Focal nodular synovitis is a benign synovial neoplasm commonly occurring around the small joints of the hands and feet. They represent the localised form of pigmented villonodular synovitis (PVNS), which usually occurs around larger joints, particularly the knee [11]. Histologically focal nodular synovitis can be confused with PVNS and GCTTS. All three entities are characterised by multinucleated giant cells and macrophages with varying degrees of haemosiderin deposition, but merely represent various forms of synovial proliferation [12]. Presence of haemosiderin results in susceptibility artefacts on GRE sequence in all three entities, but is usually most extensive in PVNS, which also shows frond-like projections into the joint. On MR, focal nodular synovitis is usually well defined, isointense or slightly hyperintense to muscle on T1w sequence and of low signal on T2w sequence because of collageneous stroma (Fig. 4). In larger lesions, variable signal on T2w sequence and heterogeneous enhancement may be seen because of proliferating capillaries [13].

### Nodular fasciitis

**Fig. 5:**
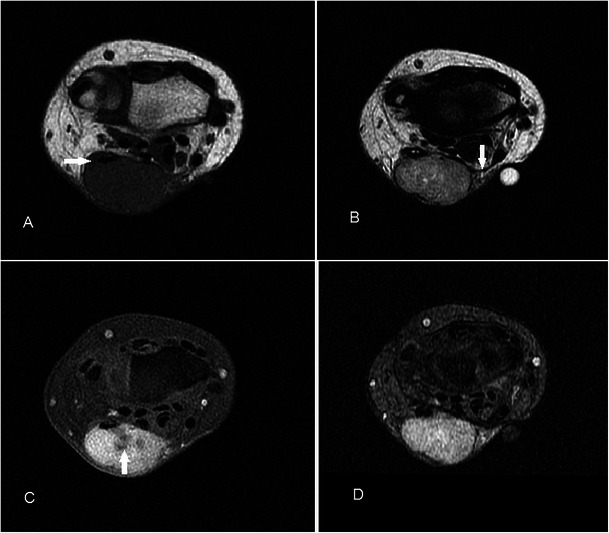
Nodular fasciitis in a 39-year-old female presenting with a rapidly enlarging left wrist lump over 2 months. (**a**) MR shows a well-circumscribed lobulated subcutaneous mass along the volar aspect of the wrist of intermediate signal on T1w sequence encasing the flexor carpi ulnaris tendon (arrow). (**b**) The mass was heterogeneously hyperintense on T2w sequence and also abuts the flexor retinaculum (arrow). (**c**) There was heterogeneous enhancement with a central non-enhancing area (arrow). (**d**) No susceptibility is detected on the GRE sequence (helps differentiate from giant cell tumour of the tendon sheath)

Nodular fasciitis is a benign reactive fibroblastic lesion usually in young adults, thought to be associated with trauma, most commonly seen in the upper extremities [14]. On MRI, it shows low signal on T1w sequence and heterogeneous, mixed high signal on T2w sequence [15], depending on the distribution of myxoid and fibrous components (Fig. 5). Lesions with predominantly cellular content or myxoid degeneration appear hyperintense on T2w sequence while those with highly collagenous contents are hypointense. Contrast enhancement pattern is most commonly diffuse but it may also be peripheral in lesions with cystic degeneration [16]. Nodular fasciitis can be easily confused with a malignant lesion given the clinical presentation of a rapidly growing lump and imaging findings of inhomogeneous signal characteristics and enhancement, lobulated borders and crossing of compartments. Differential diagnoses include neurogenic tumour, soft tissue sarcoma or early stage of myositis ossificans especially when it occurs in an intramuscular location [17]. Complete local excision is usually curative and hence careful histological analysis is necessary to avoid misdiagnosis and unnecessary radical surgery [18].

### Lipoma

**Fig. 6:**
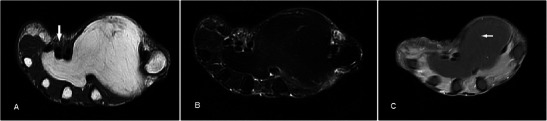
Lipoma in a 60-year-old female presenting with a soft swelling over the left thenar eminence of 1-year duration. (**a**) T1w sequence shows a well-circumscribed hyperintense lobulated subcutaneous lesion in the volar aspect of the hand involving thenar and mid-palmar spaces, insinuating between the flexor tendons (arrow). (**b**) There is homogeneous signal loss on T2w-FS sequence. (**C**) Faint enhancement of thin internal septation is noted (arrow), with no suspicious nodular component

Lipomas are the most common tumour in the human body usually developing in later adult life. A rather suggestive sonographic feature is an encapsulated hyperechoic lesion with fine linear internal echoes [19, 20]. Characteristic MR features of a well-circumscribed lesion that is hyperintense on both T1w and T2w sequences with homogeneous signal loss on the STIR or fat-saturation sequences allow confident diagnosis (Fig. 6). It can be difficult to distinguish among benign lipomas, atypical lipomatous lesions or well-differentiated liposarcomas on imaging. Findings of thick enhancing septation or a nodular component should raise suspicion of the latter [21]. Interestingly, findings of infiltrating or insinuating margins tend to suggest benign lipoma rather than liposarcoma [22].

### Lipofibromatous hamartoma

**Fig. 7:**
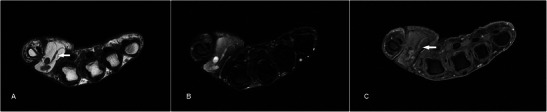
Fibrolipomatous hamartoma in a 45-year-old male presenting with a lump of 1-year duration in the first web space of the left hand. (**a**) T1w sequence shows a well-circumscribed hyperintense lobulated subcutaneous lesion within the first web space, with curvilinear low-signal structures (arrow), similar to a “spaghetti-like” appearance. (**b**) On T2w-FS sequence the lesion shows homogeneous signal loss indicating a predominantly fatty lesion while the curvilinear structures coursing through the centre of the lesion appear hyperintense. (**c**) These curvilinear structures show enhancement (arrow). These were found to represent neurovascular components of the lesion intraoperatively. The rest of the lesion does not show significant enhancement

Lipofibromatous hamartomas are rare benign tumours usually involving the median nerve at the wrist [23]. It commonly presents as a progressively enlarging lump or symptoms of compressive neuropathy, most occurring in the first 3 decades of life. Excessive fibroadipose tissue proliferates along the perineurium, surrounding the nerve bundles within the nerve sheath. This gives the lesion its pathognomic appearance on MR imaging. MR typically shows a fusiform swelling representing the enlarged nerve with serpinginous, “spaghetti” or “cable”-like appearance, representing the low-signal axons surrounded by fatty tissue [24, 25] (Fig. 7). Management is usually conservative as surgical resection is almost always accompanied by neurologic morbidity [26].

### Haemangioma

**Fig. 8:**
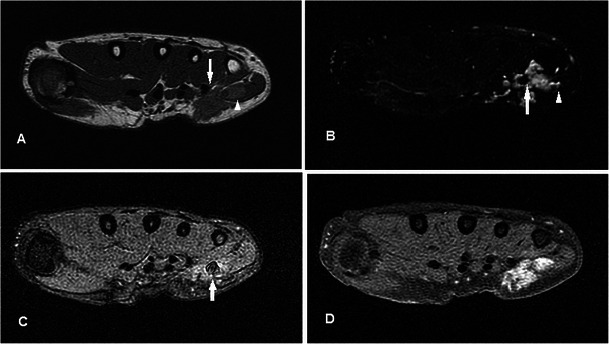
Haemangioma in a 32-year-old female presenting with a soft lump along the medial aspect of the volar aspect of right hand, occasionally painful. (**a**) T1w sequence shows a multilobulated subcutaneous lesion superficial to the hypothenar muscles slightly hyperintense to muscle, insinuating between adjacent flexor tendons (arrow). There is suggestion of a small intramuscular component (arrowhead). (**b**) The lesion is extremely hyperintense on T2w-FS sequence with areas of low signal (arrow) and suggestion of fluid-fluid level (arrowhead). (**c**) Susceptibility artefacts are seen on GRE sequence indicating phleboliths or blood products (arrow). (**d**) There is avid and near-homogeneous enhancement (arrow)

Haemangiomas are the fourth most common hand tumour, usually occuring in the younger age group, with a slight female predominance [27]. On MR, they are typically very hyperintense on T2w sequence and show lobulations, septations or low-signal foci much more frequently than other soft tissue masses [28]. Marked hyperintensity of the lesions on T2w sequence is due to increased fluid content secondary to stagnant blood flow in vessels. Signal on T1w sequence is usually iso- to hyperintense compared to muscle because of the presence of fat and blood products (Fig. 8). Lesions that are typically larger than 2 cm contain various amounts of fat, smooth muscle, myxoid stroma, thrombi and haemosiderin. Susceptibility artefacts on GRE sequence may be due to phleboliths or blood products, and these can be distinguished on plain radiograph. Larger lesions may also demonstrate fluid-fluid levels [29]. Enhancement on post-contrast imaging can be variable from minimal to heterogeneous to more homogeneous. Superficial haemangiomas are easy to diagnose because of the presence of skin discolouration and MRI may only be needed to assess their extent for surgical planning.

### Aneurysm/pseudoaneurysm

**Fig. 9:**
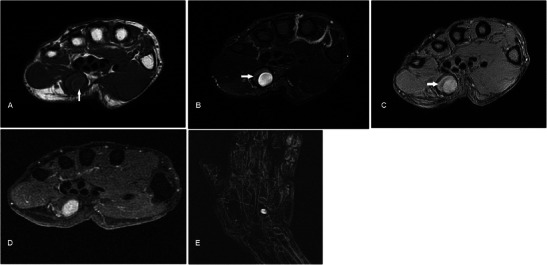
Aneurysm/pseudoaneurysm in a 32-year-old female presenting with a lump over the palmar aspect of the right hand over 4 years. There was no prior invasive medical procedure although a vague history of trauma was obtained. (**a**) T1w sequence shows a well-circumscribed subcutaneous lesion with a central, relatively high signal (arrow) located superficial to the flexor tendons. (**b**) On T2w-FS sequence the lesion is hyperintense centrally with low signal at the periphery (arrow). (**c**) The low signal periphery corresponds to susceptibility artefacts on GRE sequence (arrow), compatible with thrombosis. (**d**) There is intense enhancement centrally. The peripheral thrombus is seen as a non-enhancing area. (**e**) MR angiogram shows the lesion to be arising from the region of the distal ulnar artery

Aneurysms/pseudoaneurysms are rare lesions, usually secondary to trauma-related intimal injury, or iatrogenic from arterial punctures or arteriovenous shunts for dialysis [30]. True aneurysms can rarely occur secondary to vasculitis. MR features are variable depending on presence of thrombus or turbulent flow. They are generally slightly hyperintense on T1w and T2w sequences with areas of signal void reflecting high flow, and susceptibility artefacts may be seen on GRE sequence if there is thrombosis (Fig. 9). Either MR or conventional angiography can demonstrate continuity with a parent artery, essentially excluding other differential diagnoses [31]. Imaging features are highly suggestive even in the presence of a thrombus and allow avoiding catastrophic biopsies.

### Schwannoma

**Fig. 10:**
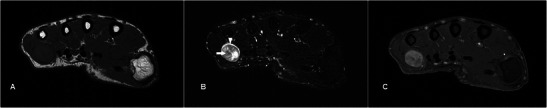
Schwannoma in a 40-year-old male with a 15-year history of non-enlarging swelling over the right palm hypothenar eminence, associated with occasional sharp pains and paresthesia. (**a**) T1w sequence shows a smooth well-circumscribed nodule within the hypothenar musculature iso-intense to adjacent muscle. (**b**) On T2w-FS sequence the lesion shows a typical target-sign appearance with a hypointense centre (arrow) and peripheral hyperintense rim (arrowhead). (**c**) Fairly homogeneous enhancement is seen

Schwannomas are the most common benign tumour of the peripheral nerve sheath, usually occuring in the 4th to 6th decades. On MR schwannomas are generally well circumscribed, show low-to-intermediate signal on T1w sequence, high signal on T2w sequence and homogeneous contrast enhancement (Fig. 10). It is often difficult to distinguish schwannomas from neurofibromas, vascular lesions or even soft tissue sarcomas. Its close relation to an expected course of a major nerve may suggest the diagnosis [32]. Larger tumours may demonstrate a dural tail, “split-fat sign” (peripheral rim of perineural fat compressed by the tumour), “fascicular sign” (central small ring-like structures representing nerve fibres) or “target sign” (central low signal with surrounding high signal on T2-weighted sequence) [33]. Schwannomas can undergo cystic or fatty degeneration. Malignant change is very rare.

## Intermediate-grade lesions

### Neurofibroma

**Fig. 11:**
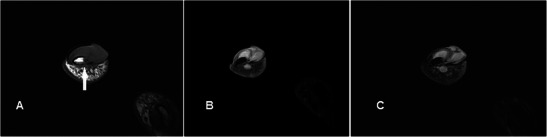
Neurofibroma in a 53-year-old male with a painless left thumb nailbed swelling of 4–5-year duration. The patient had no personal or family history of neurofibromatosis or any other clinical manifestations, and this was likely an isolated lesion. (**a**) T1w sequence shows a lobulated subcutaneous lesion at the tip of the thumb, isointense to muscle involving the skin, with mild pressure erosion on the underlying distal phalanx (arrow). (**b**) The nodule is heterogeneously hyperintense on T2w-FS sequence. (**c**) Fairly homogeneous enhancement is seen

Neurofibromas are common benign peripheral nerve sheath tumours, occurring in isolation or in relation to neurofibromatosis type 1. On MR imaging, superficial neurofibromas tend to be asymmetric, lack fascicular morphology and target-like signal intensity, and are likely to involve skin [34], features that are evident in our case (Fig. 11). They are hypo- to isointense to muscle on T1w sequence and heterogeneously hyperintense on T2w sequence [35, 36]. Neurofibromas have a propensity for recurrence as complete resection, which would require sacrificing the whole nerve, is usually not possible [34].

### Desmoplastic fibroblastoma (collagenous fibroma)

**Fig. 12:**
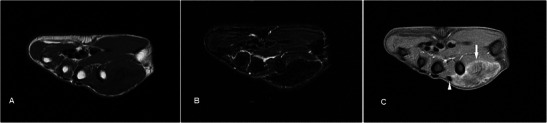
Desmoplastic fibroblastoma in a 40-year-old female who presented with a swelling of 3-month duration over the dorsum of the first web space of the right hand. (**a**) T1w sequence shows a lobulated lesion of very low signal within the first dorsal interosseous muscle with loss of surrounding fat planes. (**b**) The lesion is also of low signal on T2w-FS sequence reflecting a dense fibrous component (arrow). (**c**) There is heterogeneous predominantly peripheral enhancement (arrow). The lesion encases the index finger extensor tendon (arrowhead)

Desmoplastic fibroblastomas are rare benign myofibroblastic tumours arising in the subcutaneous tissue or skeletal muscle. There is a male predominance usually around the 5th decade. On MR these are usually of low-to-very-low signal intensity on T1w and T2w sequences and show only minimal contrast enhancement, reflecting their collagenous nature and low vascularity. Small intermixed areas of T2 hyperintensity may be seen depending upon the amount of cellular components [37]. They are only minimally infiltrative, and the presence of calcifications and cystic changes are unusual. One specific MR characteristic reported in the literature that was also seen in our case is the rim enhancement of the capsule [38, 39] (Fig. 12). Collagenous fibromas are often confused with desmoid tumours on imaging. However, desmoid tumours are often painful and more infiltrative at presentation. Moreover, desmoid tumours show prominent areas of high signal on T2w sequence and internal enhancement because of their cellular nature. Preoperative differentiation is important to avoid overtreatment and unnecessary extensive procedures performed for desmoid tumours. Other differential diagnoses include FTS, calcifying fibrous tumours, leiomyoma and GCTTS.

### Giant cell tumour of tendon sheath

**Fig. 13:**
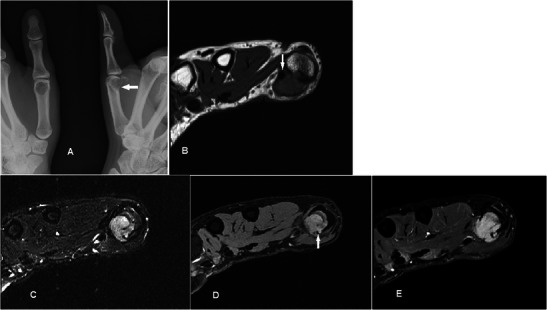
GCTTS in a 23-year-old female presenting with a 1-year history of right thumb swelling and mild pain. (**a**) Plain radiograph shows well-defined lucency at the head of the first metacarpal (arrow). (**b**) T1w sequence shows a lobulated subcutaneous lesion of intermediate signal encasing the flexor pollicis longus tendon (arrow). (**c**) The lesion shows heterogeneous high signal on T2w-FS sequence. There is pressure erosion on the underlying bone. (**d**) Foci of susceptibility are demonstrated on the GRE sequence (arrow). (**e**) There is avid and fairly homogeneous enhancement

GCTTSs are common tumours, usually presenting as painless masses at 30–50 years, with a slight female predilection. They are typically found in the hands or feet associated with degenerative joints and thought to be reactive lesions to adjacent inflammation rather than true neoplasms [40]. GCTTSs are histologically benign but pressure changes in adjacent bone can be seen on plain radiographs in 10-20 % of cases [41] (Fig. 13a). MR shows a lesion in close relation to joints and tendons, predominantly of low signal on T1w sequence and of intermediate to slightly high signal on T2w sequence (Fig. 13b-e). Susceptibility artefacts on GRE sequence are typical because of haemosiderin deposition and this is a helpful feature [42]. Strong enhancement is seen because of the presence of numerous proliferative capillaries in the collagenous stroma. Differential diagnoses include focal nodular synovitis, which also contains haemosiderin while a more heterogeneous signal and enhancement can result in confusion with soft tissue sarcomas.

### Glomus tumour

**Fig. 14:**
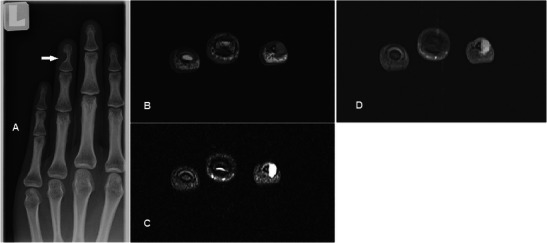
Glomus tumour in a 30-year-old female with a painful lump at the tip of the left ring finger. (**a**) Plain radiograph shows well-defined scalloping along the ulnar aspect of the left ring finger distal phalanx (arrow). (**b**) T1w sequence revealed a smooth, well-circumscribed subcutaneous lesion causing bony pressure erosion at the tip of the finger of intermediate signal. (**c**) T2w-FS sequence shows the lesion to be extremely and homogeneously hyperintense. (**d**) Intense and homogeneous enhancement is seen

Glomus tumours are benign disordered proliferation of the neuromyoarterial apparatus that serves to regulate skin circulation [43]. They usually occur in the 4th to 5th decade and are three times more frequent in females. Patients with glomus tumour seek medical attention early, but the mass is frequently too small to be identified on physical examination. The classic triad of moderate pain, temperature sensitivity and point tenderness is inconsistently present [44]. Useful distinguishing features on MR are its characteristic location, pressure erosion of underlying bone, very high and homogeneous signal on T2w sequence, low signal on T1w sequence and intense enhancement [45] (Fig. 14). Although these MR signal characteristics can be associated with any vascular tumour, the typical subungual location and its small size should lead one to suspect glomus tumour in most cases. In particular, T2w-FS and post-contrast sequences are very helpful in delineating small tumours. The lesion is typically painful, and surgery is the treatment of choice for symptom relief and histological confirmation.

## Malignant lesions

### Undifferentiated pleomorphic sarcoma

**Fig. 15:**
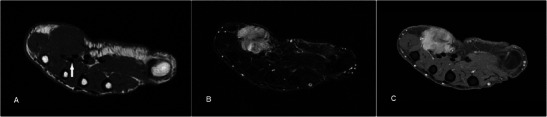
Undifferentiated pleomorphic sarcoma in a 50-year-old female presenting with a 3-month history of a firm, fixed, enlarging lump over the hypothenar eminence of the right hand. (**a**) T1w sequence shows a hypointense, predominantly subcutaneous lesion with irregular margins. It abuts flexor tendons of the little and ring fingers (arrow) and involves overlying dermis, with small areas of infiltration into underlying muscle. (**b**) The lesion is heterogeneously hyperintense on T2w-FS sequence. (**c**) Heterogeneous enhancement is demonstrated

Previously known as malignant fibrous histocytoma, this is the most common soft tissue sarcoma in adults with a wide age range of 30–80 years old and a slight male predilection. It is thought to arise from undifferentiated mesenchymal stem cells and usually occurs in the soft tissues of the retroperitoneum and proximal extremities [46]. Occurrence in the hand is rare. MR is the imaging modality of choice to stage and characterise the tumour. It is typically intermediate to low signal on T1w sequence and heterogeneously high signal on T2w sequence; however, appearance may vary depending on the presence of calcification, fibrous tissue, haemorrhage or necrosis (Fig. 15). The tumour is fairly well defined despite its malignant nature because of a pseudocapsule, but may exert a mass effect or encase neurovascular bundles. Other less common subtypes of pleomorphic sarcoma have been identified based on the proportion of myxoid, fibrous or cellular components [47].

### Primary squamous cell carcinoma of the skin

**Fig. 16:**
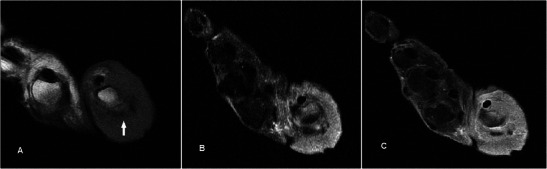
SCC in an 84-year-old female presenting with diffuse circumferential swelling over the base of the right thumb associated with ulcerations. (**a**) T1w sequence shows infiltrative circumferential skin and subcutaneous soft tissue thickening around the thumb metacarpophalangeal joint of intermediate signal. There is involvement of the extensor pollicis brevis and abductor pollicis brevis (arrow) with erosion of underlying bone. (**b**) The lesion is heterogeneously hyperintense on T2w-FS sequence. (**c**) There is also heterogeneous diffuse, circumferential enhancement

**Fig. 17:**
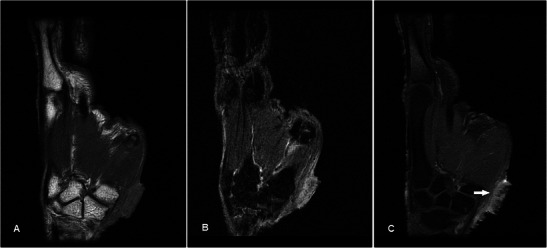
SCC in a 76-year-old female presenting with a focal ulcerating skin lump along the radial aspect of the right thumb base. (**a**) T1w sequence shows an irregular, ill-defined exophytic skin lesion at the base of thumb, of intermediate signal. (**b**) The lesion is heterogeneously hyperintense on STIR sequence. (**c**) Heterogeneous enhancement is seen with the lesion extending to the subdermis (arrow)

Primary squamous cell carcinoma (SCC) of the skin usually occurs in the older age group commonly over the sun-exposed back of hands [48]. Diagnosis is usually suspected clinically and easily confirmed on bed-side punch biopsy. Imaging is utilised for local staging. On MR, the tumour has an infiltrative appearance and can be diffuse (Fig. 16) or focal (Fig. 17). It is usually of low to intermediate signal on T1w sequence and heterogeneously hyperintense on T2w sequence with variable enhancement. Its epicentre in the cutaneous layer, irregular and infiltrative appearance should raise suspicion for a primary skin malignancy.

### Malignant bony lesions

**Fig. 18:**
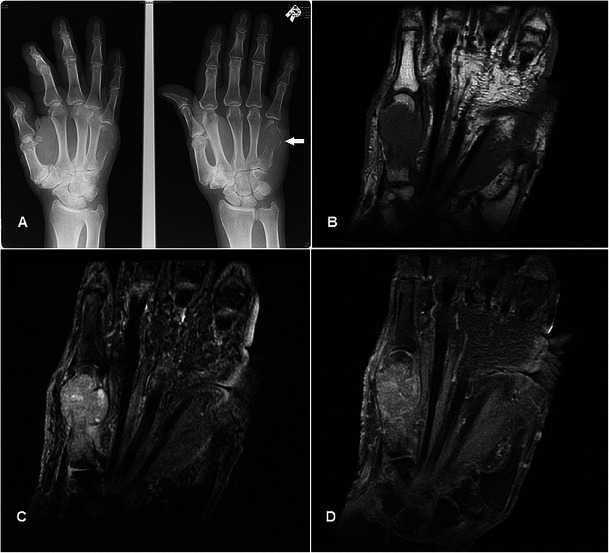
Bony metastasis in a 67-year-old male with a history of adenocarcinoma of the lung, presenting with a 2-week history of swelling and pain over the medial aspect of the right hand dorsum. (**a**) Plain radiograph shows permeative destruction of the fifth metacarpal (arrow). (**b**) T1w sequence shows destruction of the fifth metacarpal with infiltrative soft tissue, predominantly of heterogeneous intermediate signal. (**c**) The lesion is heterogeneously hyperintense on T2w-FS sequence. (**d**) Heterogeneous enhancement is seen extending to the adjacent soft tissues

Osseous metastases to the hand (acrometastases) is uncommon. The most common primary sites of malignancies are the lung, kidney and breast, and they usually indicate poor prognosis [49]. Plain radiographs showing expansile destructive lytic lesions are usually diagnostic in the appropriate setting (Fig. 18a). MR can be useful if there is any uncertainty about the clinical history or unusual radiographic appearance (Fig. 18b-d). Treatment is palliative but can improve quality of life.

**Fig. 19:**
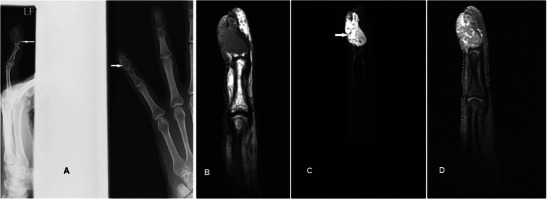
Low-grade chondrosarcoma in a 47-year-old female presenting with a slowly enlarging firm swelling over the distal phalanx of the left little finger for several months with nail deformity. (**a**) Plain radiograph shows an expansile lytic bony lesion in the distal phalanx of the little finger with marked endosteal scalloping and thinning with disruption of the dorsal cortex. Ring-and-arc densities are suggestive of chondroid matrix (arrows). (**b**) T1w sequence shows an expansile bony lesion in the little finger distal phalanx of intermediate signal, with a soft tissue component destroying and breaking through the dorsal cortex to involve the skin and nailbed. (**c**) The lesion is very hyperintense on T2w-FS sequence with hypointense areas (arrow), typical of a chondroid matrix. (**d**) Post-contrast there is heterogeneous enhancement

Chondrosarcoma is the most common primary bone malignancy in adults and may occur as a malignant degeneration of a benign chondroid lesion such as an enchondroma. Plain radiograph shows a typical “ring-and-arc” matrix with endosteal scalloping and cortical thinning, but in higher grade subtypes there is often bony destruction and irregular margins [50] (Fig. 19a). MR is usually used to for local staging (Fig. 19b-d). Differential diagnosis for a lesion in the distal phalanx is a glomus tumour, but the plain radiograph showing a primarily expansile bony lesion effectively excludes it.

### Fibrosarcoma of the tendon sheath

**Fig. 20:**
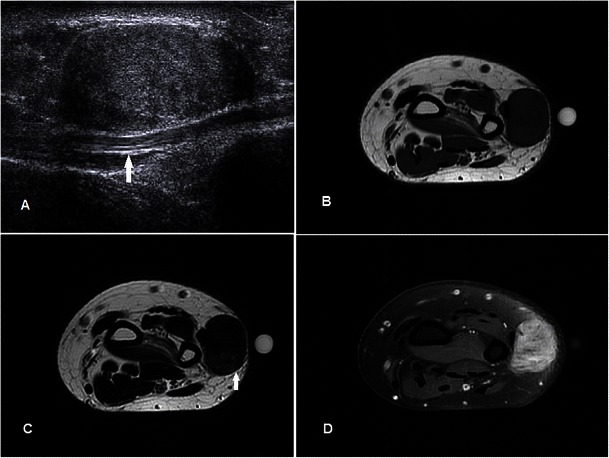
Fibrosarcoma of the tendon sheath in a 47-year-old female presenting with focal swelling along the ulna aspect of the right wrist, gradually increasing in size over a few months. (**a**) Ultrasound shows a well-defined hypoechoeic lesion along the ulnar side of the wrist abutting the extensor carpi ulnaris (arrow). (**b**) T1w sequence shows a hypointense well-circumscribed subcutaneous lesion abutting the extensor carpi ulnaris (arrow). (**c**) The lesion is predominantly of low signal on T2w sequence, with small areas of high signal (arrow) indicating a more cellular/necrotic component. (**d**) Heterogeneous enhancement is seen, in contrast to a benign FTS (Fig. 3), which shows only minimal enhancement

Fibrosarcoma is a rare malignancy of mesenchymal origin composed of fibroblasts and collageneous matrix. The primary adult form is slightly more common in males in the 35–55-year age group. They usually arise from the joint capsule or bones around the knee and pelvis, less commonly from soft tissue such as muscle or the tendon sheath [51]. Secondary forms occur in a setting of prior irradiation or other malignant transformation from benign bony disorders such as Paget’s disease. On MR, the lesion is usually of low signal on both T1w and T2w sequences reflecting a fibrous matrix but may be interspersed with high-signal areas on T2w sequence from increased cellularity or necrosis (Fig. 20). Post-contrast there is usually intense enhancement, and a “spoke-wheel” pattern of enhancement has been described [52]. Necrosis and haemorrhage commonly seen in high-grade fibrosarcomas is uncommon in the low-grade variety. Differentiating low-grade fibrosarcomas from fibromatosis and its variants may be difficult. Histological analysis is required for the definite diagnosis.

## Pseudo-masses

### Inflammatory pseudotumour

**Fig. 21:**
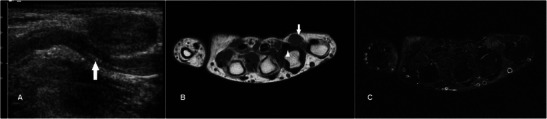
Inflammatory pseudotumour in a 50-year-old female presenting with a painless mobile lump over the medial aspect of the palm for a few months. (**a**) Ultrasound shows a solid heterogeneous lesion in the palm over the fourth web space close to the flexor tendon of the ring finger (arrow). (**b**) T1w sequence shows a lobulated subcutaneous nodule of intermediate signal (arrow) close to but separate from the flexor tendon of ring finger (arrowhead). (**d**) The lesion is only minimally hyperintense on T2w-FS sequence. No intravenous contrast was given because of renal impairment

Inflammatory pseudotumours are thought to be an abnormal inflammatory response to trauma or an infectious agent [53]. They occur over a wide age range with equal male to female distribution. Usually seen in the orbits, head and neck, lung and various sites in the abdomen, their occurrence in the hand is rare [54]. MR features are non-specific, usually those of a well-circumscribed lesion of low signal on T1w sequence and variable signal on T2w sequence with variable enhancement (Fig. 21). Although this lesion is considered benign, recurrence and malignant change have been reported and complete surgical resection is required. Histologically, this entity encompasses a spectrum from early inflammatory lesions to chronic calcifying/sclerotic ones [55].

### Gout

**Fig. 22:**
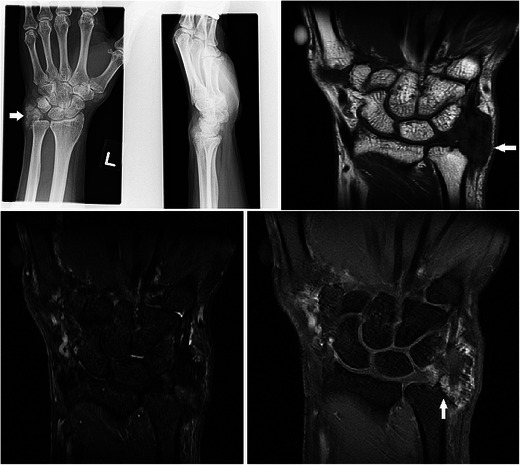
Gouty tophus in a 60-year-old male with known gout, presenting with a 6-month duration of gradual swelling along the ulna aspect of the left wrist. (**a**) Plain radiograph shows amorphous calcification adjacent to the ulna styloid (arrow). (**b**) T1w sequence shows an irregular lobulated mass of low signal (arrow). (**c**) The lesion was also hypointense on T2w-FS sequence. (**d**) Mild peripheral enhancement is seen, and there were also erosions of the underlying ulna styloid (arrow)

Gouty tophi can present as focal masses. Plain radiographs usually suggest the diagnosis. Juxta-articular erosions with overhanging edges and associated calcified soft tissue masses are typical findings (Fig. 22a). MR findings of gouty tophi are also rather characteristic. The lesions are of low to intermediate signal on all MR sequences mainly because of the presence of calcification and can show peripheral enhancement [56] (Fig. 22b-d).

### Tendon abnormalities

**Fig. 23:**
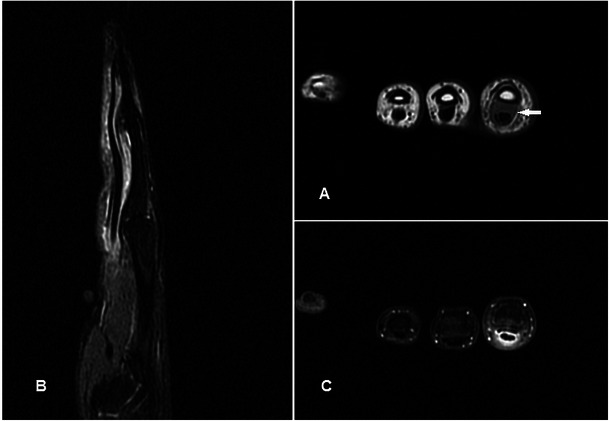
Tenosynovitis in a 24-year-old female presenting with 1-month duration of swelling, pain and loss of range of motion over the flexor aspect of the left index finger. (**a**) T1w sequence shows diffuse thickening of the index finger flexor tendon sheath (arrow). (**b**) T2w-FS sequence shows thickening and increased signal of the flexor tendon sheath indicated oedema. (**c**) Diffuse enhancement of the sheath is seen

Tenosynovitis refers to inflammation of the tendon and tendon sheath. Localised inflammation can appear mass-like clinically. MRI findings of tenosynovitis include increased fluid signal within the tendon sheath, tendon sheath distension, synovial proliferation and enhancement (Fig. 23). According to one study, flexor tenosynovitis of the hand diagnosed by MRI is a strong predictor of early rheumatoid arthritis [57].

**Fig. 24:**
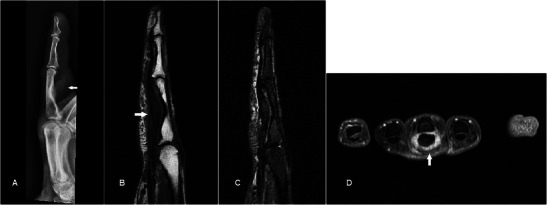
Chronic calcific tendinopathy in a patient presenting with 10-year history of focal swelling along the volar aspect of the left middle finger.(**a**) Plain radiograph shows well-defined scalloping along the volar aspect of the middle finger proximal phalanx with an overlying striated pattern of calcification (arrow). (**b**) T1w sequence shows focal swelling of the flexor digitorum longus tendon (arrow) causing scalloping of the underlying bone. No discrete mass is seen. (**c**) There is a mild increase in signal in the tendon on T2-FS sequence suggesting tendinosis. There is bony scalloping but no significant marrow oedema, suggesting a chronic process. (**d**) Diffuse thickening and intense enhancement of the tendon sheath is seen, reflecting an element of active tenosynovitis (arrow)

In unusual cases of chronic tendinopathy, chronic inflammation and swelling of a tendon presented as focal swelling resulting in bony scalloping and a striated pattern of calcification (Fig. 24). Main differential diagnosis in this case is a GCTTS but MR effectively excludes an underlying mass lesion.

## Discussion

MR characteristics of various soft tissue lesions in the hand are well described; however, preoperative diagnosis is difficult as imaging features are usually non-specific and features of different lesions may overlap. Certain features on MR can suggest the nature of the lesion. For example, vascular lesions tend to have slightly increased signal on T1w sequence, fatty lesions are hyperintense on both T1w and T1w sequences, while fibrous lesions tend to demonstrate low signal on all pulse sequences. The location of a lesion based on its relation to the carpi, metacarpals or phalanges can also help limit differential diagnoses [58]. Several studies have attempted to distinguish benign from malignant lesions on imaging. For example, benign lesions tend to have homogeneous signal and well-defined margins while malignant soft tissue lesions tend to demonstrate less well-defined margins, lobulation, fascial oedema, haemorrhage and necrosis (hence heterogeneous signal and enhancement) [59, 60]. Larger and deep-seated tumours have been shown to be associated with increased signal heterogeneity and hence likelihood of malignancy [61]. However, this has been countered by Chung et al., who found that 43 % of histologically proven benign soft tissue tumours were >5 cm in size and 57 % were deeply located beneath the superficial fascia [62]. Despite these findings in the literature, differentiating benign from malignant soft tissue lesions preoperatively remains difficult as most soft tissue lesions, benign or malignant, often demonstrate smooth borders and homogeneous MR signal [63]. A classification based on MR imaging features would be more helpful for management as well as prognosis in cases of malignant soft tissue lesions. Established staging systems for extremity soft tissue sarcomas involve three parameters of size, depth and histological grade, and it is found that the staging system that incorporates all three parameters (Memorial Sloan-Kettering Cancer Centre Staging System) was the best predictor of relapse [64]. In addition, the Surgical Staging System (SSS) classifies soft tissue sarcomas based on compartmental status [65]. Although the hand is thought to be poorly compartmentalised, the subfascial spaces can be divided into five main compartments: the mid-palmar, thenar and hypothenar spaces ventrally, the dorsal subaponeurotic space deep to the extensor tendons and superficial to the metacarpals, and the space of Parona around the wrist, superficial to the pronator quadratus and interosseus membrane and deep to the flexor digitorum profundus and superficialis tendons [66]. SSS utilises a more practical approach to compartmental status. In the hand, intra-compartmental lesions lie close to or involve the bony structures, digital soft tissues, extensor and flexor tendons and intrinsic musculature, while extra-compartmental lesions involve the nerves, vasculature and subcutaneous soft tissues. For malignant lesions extracompartmental involvement indicates at least SSS stage IB, IIB or IIIB disease [67] and this classification enables stage-appropriate management.

We propose a classification of hand lesions in our review (excluding the pseudo-masses) into “benign”, “intermediate grade” (histologically benign but locally aggressive with potential for recurrence) and “malignant” lesions. This is based on specific MR features in terms of signal, enhancement, lesion margins, presence of bony destruction and compartmental involvement (Table 1).

**Table 1:** Proposed classification of focal hand lesions at our institution into benign, intermediate-grade and malignant lesions

Lesion	Margins	T1w signal	T2w signal	Crossing compartments/involvement of adjacent structures	Bony destruction	Enhancement pattern
Benign						
Ganglion cyst	Smooth	Homogeneously hypointense	Homogeneously hyperintense (fluid)	Nil	NA	Rim enhancement, no internal enhancement
Epidermal cyst	Smooth	Homogeneously hypointense	Homogeneous hyperintense (fluid)	Nil	NA	Rim enhancement, no internal enhancement
Fibroma of tendon sheath	Lobulated, preserved fat planes	Intermediate	Heterogeneously hyperintense with low signal areas	Nil	Nil	Small eccentric focus of enhancement
Focal nodular synovitis	Fairly smooth	Hypointense	Homogeneously hypointense	Nil	Nil	No significant enhancement
Nodular fasciitis	Lobulated	Intermediate	Heterogeneously hyperintense	Crosses compartments (intra-and extra-compartmental)	NA	Heterogeneous, central non-enhancing foci
Lipoma	Lobulated, insinuating margins, preserved fat planes	Hyperintense	Homogeneous fat signal intensity	Crosses compartments (intra-and extra-compartmental, involves thenar and mid-palmar spaces)	Nil	Enhancement of thin internal septae only
Lipofibromatous hamartoma	Lobulated	Hyperintense with curvilinear low signal structures within	Homogeneous fat signal intensity surrounding neurovascular component	Nil	Nil	Linear enhancement of neurovascular components within the lesion
Haemangioma	Lobulated, insinuating margins	Slightly hyperintense	Heterogeneously hyperintense	Crosses compartments (intra-and extra-compartmental)	Nil	Avid, near homogenous post contrast enhancement
Schwannoma	Smooth	Isointense (to muscle)	Heterogeneously hyperintense, target sign	Nil	NA	Avid, near homogenous
Intermediate-grade (histologically benign but locally aggressive with potential for recurrence)						
Neurofibroma	Lobulated	Isointense	Heterogeneous	Crosses compartments (intra-and extra-compartmental)	Pressure erosion	Avid, near homogeneous
Desmoplastic fibroblastoma	Lobulated, loss of fat planes	Hypointense	Heterogeneous low signal	Encases adjacent extensor tendon	Nil	Heterogeneous internal and capsule enhancement
GCTTS	Lobulated	Intermediate	Heterogeneous intermediate-high with low signal areas due to haemosiderin deposits	Crosses compartments (intra-and extra-compartmental)	Pressure erosion	Avid, near homogeneous
Glomus tumour	Smooth	Intermediate	Homogeneously hyperintense	Crosses compartments (intra-and extra-compartmental)	Pressure erosion	Avid, homogenous
Malignant						
Undifferentiated pleomorphic sarcoma	Irregular	Hypointense	Heterogeneously hyperintense	Crosses compartments (intra-and extra-compartmental, areas of infiltration into adjacent muscle)	Nil	Heterogeneous
SCC	Infiltrative	Hypointense/intermediate	Heterogeneously hyperintense	Invading adjacent muscle, skin, bone	Yes	Heterogeneous
Malignant bone lesions	Infiltrative	Hypoointense/intermediate	Heterogeneously hyperintense	Invading adjacent muscle, skin, bone, soft tissue component	Yes	Heterogeneous
Fibrosarcoma	Fairly well defined	Hypointense	Heterogeneously intermediate to low signal	Nil (extracompartmental)	Nil	Heterogeneous, small non-enhancing foci

The most consistent observation from our review is that lesions classified as benign often do not show significant internal enhancement. Those that enhance (such as haemangiomas, schwannomas) tend to be homogeneous. They also usually demonstrate smooth margins and homogeneous signal on T2w sequence while intermediate-grade and malignant lesions tend to show more heterogeneous signal. Larger benign lesions may show lobulated margins, insinuate around adjacent structures or cross compartments (such as large lipomas), resembling intermediate-grade lesions in this respect. However, the surrounding fat planes are preserved, while intermediate-grade lesions (such as desmoplastic fibromas) may show loss of surrounding fat planes. Bony scalloping with no overt bony destruction is suggestive of an intermediate-grade lesion (GCTTS, glomus tumours, neurofibromas). Frank bony destruction and irregular, infiltrative margins certainly suggest a malignant lesion. One exception is benign lipomas, which are more likely to demonstrate insinuating margins and crossing of compartments than liposarcomas [24]. We postulate that benign lipomas tend to be softer and hence insinuate more easily than their malignant counterpart. Another exception is that of soft tissue sarcomas. Despite their malignant nature, they may show fairly well-defined or lobulated margins and can be confused with the intermediate-grade lesions radiologically. In these situations, the pattern of enhancement can be a useful distinguishing feature. Malignant lesions tend to show heterogeneous enhancement while intermediate-grade lesions tend to enhance homogeneously. Another notable exception is that of nodular fasciitis. While this is a benign entity and almost never recurs after excision, the heterogeneous signal and enhancement on MR resembles that of a malignant lesion, such as the case of pleomorphic sarcoma. Only histological examination confirms the benign nature of this lesion and avoids more extensive surgery.

Our classification of “benign” and “malignant” lesions is also consistent with the WHO classification of soft tissue and bone tumours [68]. Although two lesions in our review classified as “intermediate-grade” lesions (GCTTS and desmoplastic fibroblastoma) are deemed “benign” in the WHO classification, we attribute this difference to the fact that our classification is based on preoperative imaging criteria, while the WHO classification is based on known histology and biological behavior.

## Conclusion

Imaging, particularly MR, plays an important role in characterisation of hand lesions. We propose a classification of these lesions into benign, intermediate-grade and malignant lesions based on MR features. Together with established classifications based on lesion depth, size and compartment status, we believe our classification will help further management of focal hand lesions in terms surgical planning and, in cases of primary malignancy, local staging and prognosis. A formal prospective study or systematic review would be useful to validate our proposed classification.
